# *Streptomyces* spp. as efficient expression system for a d,d-peptidase/d,d-carboxypeptidase involved in glycopeptide antibiotic resistance

**DOI:** 10.1186/1472-6750-13-24

**Published:** 2013-03-16

**Authors:** Elisa Binda, Giorgia Letizia Marcone, Francesca Berini, Loredano Pollegioni, Flavia Marinelli

**Affiliations:** 1 Department of Biotechnology and Life Sciences, University of Insubria, via J. H. Dunant 3, Varese, Italy; 2 “The Protein Factory” Research Center, Politecnico of Milano, ICRM CNR Milano and University of Insubria, Varese, Italy

**Keywords:** *Streptomyces*, Heterologous protein production, d,d-carboxypeptidases, Glycopeptide production, Glycopeptide resistance, Dalbavancin

## Abstract

**Background:**

VanY_n_, encoded by the *dbv7* gene (also known as *vanY*_*n*_) of the biosynthetic cluster devoted to A40926 production, is a novel protein involved in the mechanism of self-resistance in *Nonomuraea* sp. ATCC 39727. This filamentous actinomycete is an uncommon microorganism, difficult-to-handle but biotechnologically valuable since it produces the glycopeptide antibiotic A40926, which is the precursor of the second-generation dalbavancin in phase III of clinical development. In order to investigate VanY_n_ role in glycopeptide resistance in the producer actinomycete an appropriate host-vector expression system is required.

**Results:**

The cloning strategy of *vanY*_*n*_ gene (G-C ratio 73.3%) in the expression vector pIJ86 yielded a recombinant protein with a tag encoding for a histidine hexamer added at the C-terminus (C-His_6_-*vanY*_*n*_) or at the N-terminus (N-His_6_-*vanY*_*n*_). These plasmids were used to transform three *Streptomyces* spp*.*, which are genetically-treatable high G-C content Gram-positive bacteria taxonomically related to the homologous producer *Nonomuraea* sp.. Highest yield of protein expression and purification (12 mg of protein per liter of culture at 3 L bioreactor-scale) was achieved in *Streptomyces venezuelae* ATCC 10595, that is a fast growing streptomyces susceptible to glycopeptides. VanY_n_ is a transmembrane protein which was easily detached and recovered from the cell wall fraction. Purified C-His_6_-VanY_n_ showed d,d-carboxypeptidase and d,d-dipeptidase activities on synthetic analogs of bacterial peptidoglycan (PG) precursors. C-His_6_-VanY_n_ over-expression conferred glycopeptide resistance to *S. venezuelae*. On the contrary, the addition of His_6_-tag at the N-terminus of the protein abolished its biological activity either *in vitro* or *in vivo* assays.

**Conclusions:**

Heterologous expression of *vanY*_*n*_ from *Nonomuraea* sp. ATCC 39727 in *S. venezuelae* was successfully achieved and conferred the host an increased level of glycopeptide resistance. Cellular localization of recombinant VanY_n_ together with its enzymatic activity as a d,d-peptidase/d,d-carboxypeptidase agree with its role in removing the last d-Ala from the pentapeptide PG precursors and reprogramming cell wall biosynthesis, as previously reported in glycopeptide resistant pathogens.

## Background

Filamentous actinomycetes are high G-C Gram-positive microorganisms commercially widely used as producers of natural products (in particular antibiotics) and industrial enzymes [1]. Genome sequencing of representative microbes belonging to this group has shown that they possess a vast array of genes devoted to the production and secretion of enzymes, due to the role they play in recycling organic material in the biosphere [2]. Genome annotation of *Streptomyces coelicolor*[3], which is the model system for this microbial group, revealed that it encodes 819 potentially secreted proteins including hydrolases, proteases/peptidases, chitinases/chitosanases, cellulases/endoglucanases, amylases and pectate lyases. Moreover, filamentous actinomycetes are rich of novel enzymatic functionalities since each genome hosts twenty-thirty biosynthetic gene clusters (20–100 kbp each) devoted to the production of chemically diverse bioactive metabolites [1]. Among filamentous actinomycetes, the most studied genus is the *Streptomyces* one, whose members produce two-thirds of the known antibiotics [4]. *Streptomyces* spp. are also used as host systems for the production of heterologous proteins and of whole biosynthetic clusters originating from less easy-to-handle actinomycetes, such as those belonging to *Nonomuraea*, *Actinoplanes, Planomonospora, Planobispora, Microbispora* genera [1]. These uncommon actinomycetes possess a still-untapped richness of metabolic pathways - and some of them are valuable producers of new drugs - but their exploitation is often limited by the lack of genetic manipulation tools [5,6].

*Streptomyces* spp. as heterologous hosts for gene expression and protein production offer some advantages in comparison to *Escherichia coli*, the preferred cell factory for industrial enzymes and therapeutic proteins [7,8]. Production and isolation of heterologous proteins from *E. coli* is often limited by insolubility, citotoxicity, uncorrect folding, aggregation in inclusion bodies and lack of secretion [2]. Secretion capability of *Streptomyces* spp. may prevent the local accumulation of the over-expressed recombinant proteins, reduce their toxicity to host cells, eventually aid correct folding and favour increased production and purification yields [9]. Heterologous expression is often facilitated when the selected host cells are phylogeneticaly related to the homologous producer. This is due to the similarity of codon usage, compatibility with translation machinery, molecular chaperons, and/or redox state of the cells [2].

We recently started studying the role of genes involved in the biosynthesis, regulation, transport and self-resistance of glycopeptide antibiotics in producing strains which belong to uncommon actinomycetes such as *Actinoplanes teichomyceticus* producing teicoplanin [10,11] and *Nonomuraea* sp. ATCC 39727, which produces A40926 [12]. A40926 is the precursor of the semi-synthetic derivative dalbavancin, which is a second generation glycopeptide currently in phase III clinical development for its improved activity, pharmacokinetics and pharmacodynamics [13]. The *dbv* cluster, which is a contiguous set of 37 ORFs devoted to the production, regulation and transport of A40926, contains the *vanY*_*n*_ gene, whose function was proposed to confer glycopeptide resistance to the producing strain by reprogramming peptidoglycan cell wall biosynthesis [12,13].

The aim of this work was developing an appropriate heterologous expression system for VanY_n_ characterization to help deciphering its role in glycopeptide resistant cells. Our attention was given to screening different *Streptomyces* spp. as hosts for recombinant VanY_n_ secretion in biologically active form. Conditions for production (and purification) of functional VanY_n_ were finally successfully settled in *Streptomyces venezuelae* ATCC 10595 at flask and at fermentor-scale.

## Results

### Heterologous expression of VanY_n_ in *Streptomyces* spp

*vanY*_*n*_ encoding gene (CAD91202) was amplified by PCR using chromosomal DNA template from *Nonomuraea* sp. ATCC 39727 [12] and cloned in the multicopy vector pIJ86, under the control of the heterologous constitutive promoter ermE*[14]. The gene was cloned in frame with a sequence encoding for a histidine hexamer added at the C-terminus (C-His_6_-*vanY*_*n*_) or at the N-terminus (N-His_6_-*vanY*_*n*_) of the protein product.

pIJ86ΩC-His_6_-*vanY*_*n*_, pIJ86ΩN-His_6_*-vanY*_*n*_, and the empty pIJ86 used as a control, were introduced in three different *Streptomyces* spp. by intergeneric conjugation from *E. coli*. *Streptomyces lividans* TK24 was selected as one of the hosts since it is used for heterologous protein production due to its proven excellence in secretion capacity and low extracellular protease activity [15]. *Streptomyces venezuelae* ATCC 10595 is a fast growing streptomyces naturally susceptible to glycopeptides [12]. *Streptomyces coelicolor* A3(2) represents the model system [3] and possesses a complete set of *vanRSHAX* genes conferring high resistance to vancomycin: consequently a glycopeptide susceptible mutant deleted in the two component regulatory system *ΔvanRS* was used in our experiments [16].

**Figure 1:**
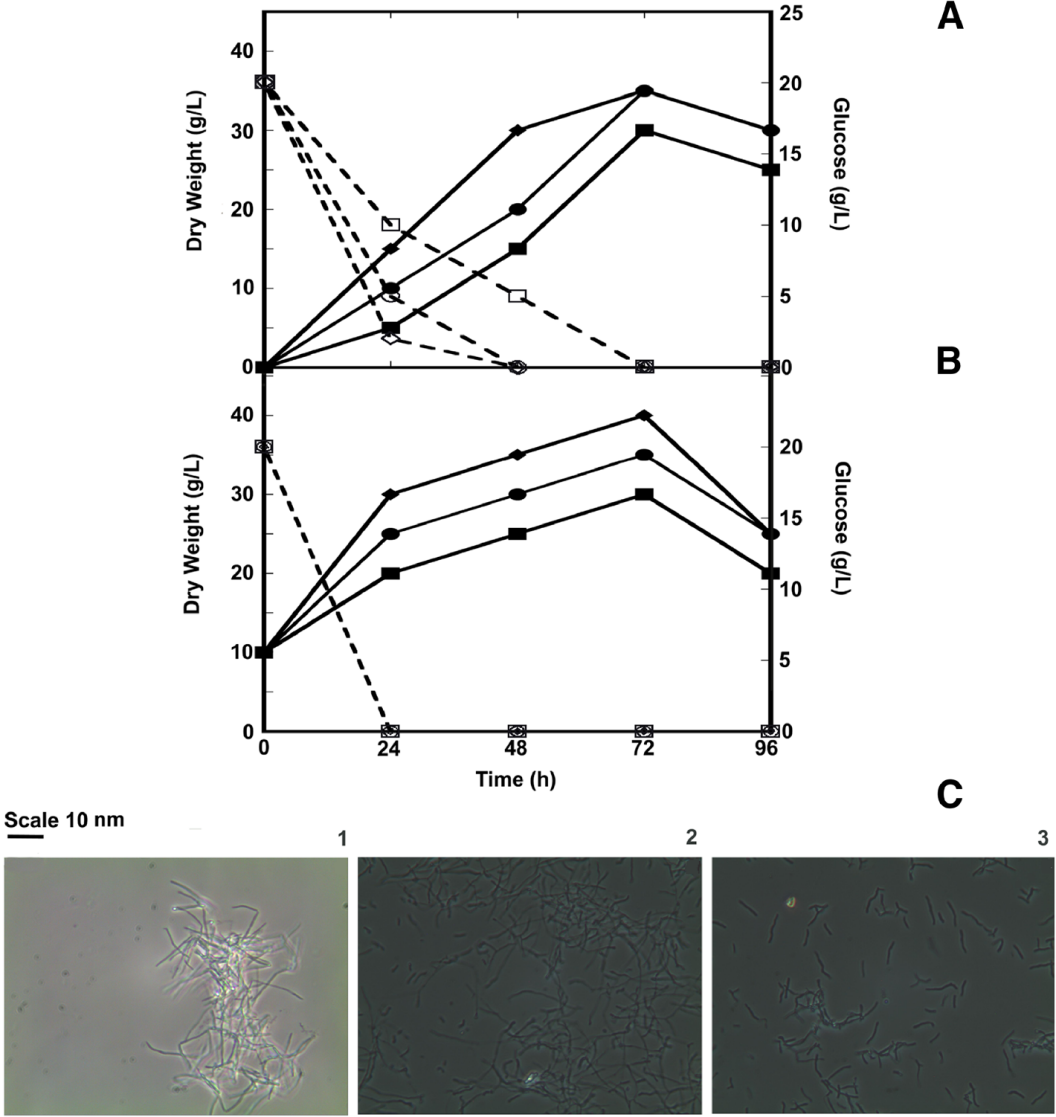
**Growth of *S. venezuelae *pIJ86ΩC-His**_**6**_**-*vanY***_***n***_**, *S. venezuelae *pIJ86ΩN-His**_**6**_**-*vanY***_***n ***_**and *S. venezuelae *pIJ86 in YEME (A) and in BTSB (B) at flask level.***S. venezuelae* pIJ86ΩC-His_6_-*vanY*_*n*_ dry weight (■ , solid line) and glucose consumption (□, dashed line); *S. venezuelae* pIJ86ΩN-His_6_-*vanY*_*n*_ dry weight (● , solid line) and glucose consumption (○, dashed line); *S. venezuelae* pIJ86 dry weight (♦, solid line) and glucose consumption (◊, dashed line). In (**C**), morphology of *S. venezuelae* pIJ86 (**1**), *S. venezuelae* pIJ86ΩN-His_6_-*vanY*_*n*_ (**2**) and *S. venezuelae* pIJ86ΩC-His_6_-*vanY*_*n*_ (**3**) grown in YEME for 72 hours at the optical microscopic observation (Zeiss Primo Star phase-contrast microscopy with 40X enlargement).

Growth curves at shake flask-scale of the ex-conjugants *S. coelicolor ΔvanRS*, *S. lividans* and *S. venezuelae* carrying pIJ86ΩC-His_6_*-vanY*_*n*_ or pIJ86ΩN-His_6_-*vanY*_*n*_ were compared with control strains containing the empty vector in two different cultivation conditions, i.e. by using the limpid YEME medium or the rich and complex BTSB medium (Figure 1 and Table 1). Figure 1A and 1B show the growth curves of *S. venezuelae* recombinant strains, which produced abundant biomass in both the media. Introduction of *vanY*_*n*_ slightly affected biomass productivity. Growth kinetics was similar among *S. venezuelae* carrying either C-His_6_-*vanY*_*n*_ or N-His_6_-*vanY*_*n*_ constructs and the control strain. Maximum biomass production (≥ 30 g/L dry weight) was achieved after 72 hours from the inoculum in both YEME and BTSB media. Glucose was consumed with different kinetics between the two media, but it was anyhow completely depleted within 72 hours of growth. Observation at the optical microscope showed that mycelium in recombinant strains was more fragmented than in the control one (Figure 1C).

**Table 1:** **Biomass and VanY**_**n **_**production in recombinant *Streptomyces *spp.**

**Strain**	**Vector**	**Medium**	**Dry weight (g cell/L)**	**VanY**_**n **_**(mg/g cell)**	**VanY**_**n **_**(mg/L)**
*S. venezuelae*	pIJ86ΩC-His_6_-*vanY*_*n*_	BTSB	30	0.94	28.4
*S. venezuelae*	pIJ86ΩC-His_6_-*vanY*_*n*_	YEME	30	0.97	30.7
*S. venezuelae*	pIJ86ΩN-His_6_-*vanY*_*n*_	BTSB	36	1.47	23.9
*S. venezuelae*	pIJ86ΩN-His_6_-*vanY*_*n*_	YEME	35	1.10	31.6
*S. coelicolor ΔvanRS*	pIJ86ΩC-His_6_-*vanY*_*n*_	BTSB	25	1.16	28.9
*S. coelicolor ΔvanRS*	pIJ86ΩC-His_6_-*vanY*_*n*_	YEME	20	0.62	12.4
*S. coelicolor ΔvanRS*	pIJ86ΩN-His_6_-*vanY*_*n*_	BTSB	30	1.06	31.8
*S. coelicolor ΔvanRS*	pIJ86ΩN-His_6_-*vanY*_*n*_	YEME	8	0.79	6.3
*S. lividans*	pIJ86ΩC-His_6_-*vanY*_*n*_	BTSB	20	1.02	20.3
*S. lividans*	pIJ86ΩN-His_6_-*vanY*_*n*_	BTSB	22	0.80	18

*Streptomyces* spp. recombinant strains carrying different constructs (C-His_6_-*vanY*_*n*_ or N-His_6_-*vanY*_*n*_) were grown in YEME or BTSB for 72 hours. VanY_n_ was quantified by immunoblotting on crude extracts as reported in the Methods.

Data in Table 1 shows that *S. coelicolor ΔvanRS* recombinant strains grew better in BTSB than in YEME, whereas recombinant *S. lividans* strains did not grow in YEME medium. Different effects of medium composition on growth rate and morphology of suspension cultures (i.e. size of mycelium pellets) among diverse streptomyces hosts have been previously observed [14]. Production of His_6_-VanY_n_ was evaluated by Western blot analysis after 72 hours of growth in *S. venezuelae, S. coelicolor ΔvanRS* and *S. lividans* recombinant strains. In BTSB medium, recombinant VanY_n_ volumetric and specific productivities ranged between 18 to more than 30 mg per liter of culture and from 0.8 to more than 1 mg per gram of cells, within the different recombinant strains. In YEME medium, the highest values of volumetric (about 30 mg of VanY_n_ per liter of culture) and specific (slightly less than 1 mg of enzyme per gram of cells) productivities were achieved only for *S. venezuelae* recombinant strains. Considering that BTSB medium contains complex components which may later on interfere with protein purification, YEME medium was preferred for large-scale protein preparation. Accordingly, *S. venezuelae* was selected as the preferable host for protein production scaling-up and purification.

**Figure 2:**
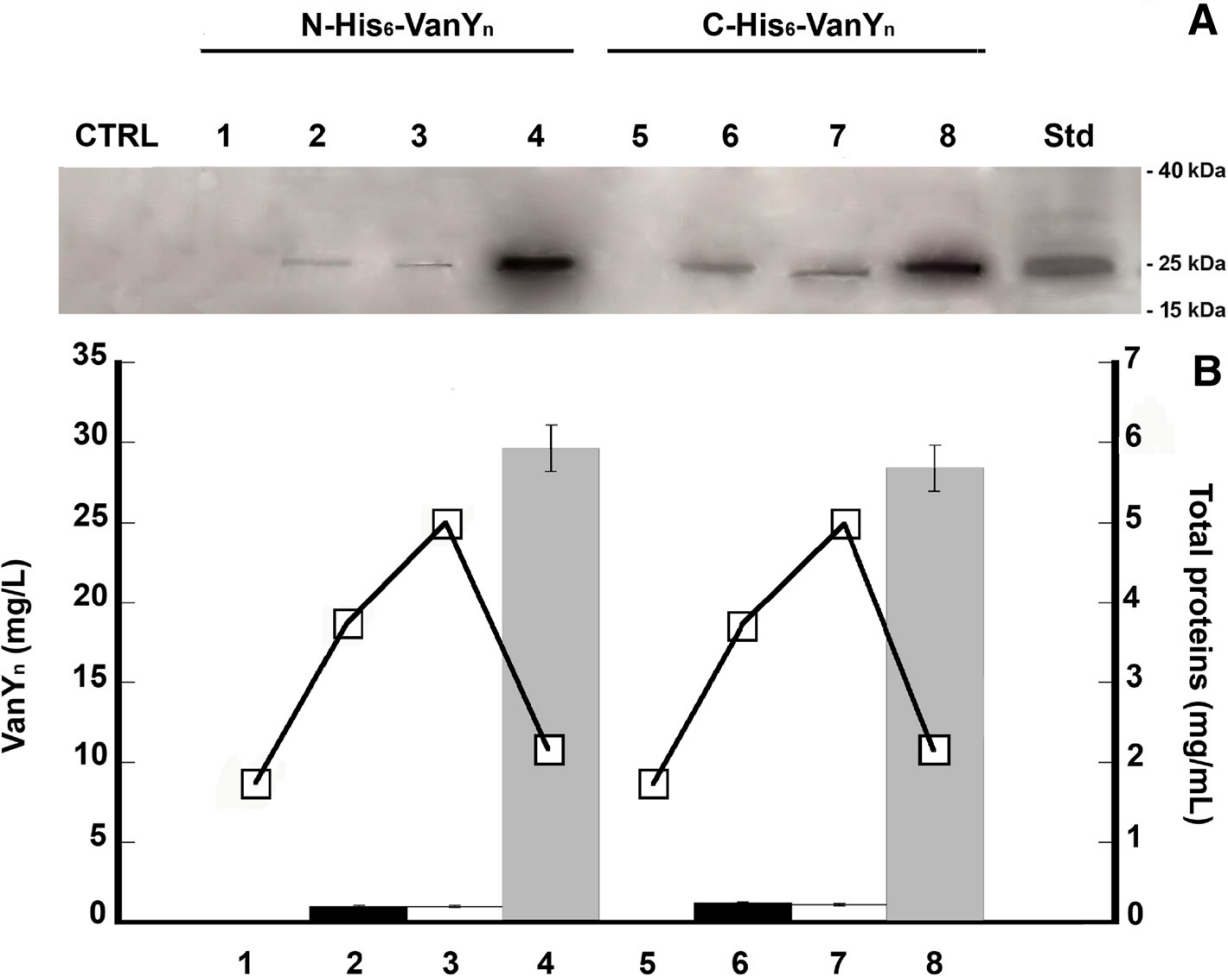
**Western blot analysis of C-His**_**6**_**-VanY**_**n **_**and N-His**_**6**_**-VanY**_**n **_**from *S. venezuelae *recombinant strains.** (**A**): analysis of samples corresponding to cellular and extracellular fractions from cultures of *S. venezuelae* recombinant strains grown in YEME for 72 hours. From *S. venezuelae* pIJ86ΩN-His_6_-*vanY*_*n*_: extracellular fraction (lane 1), soluble cell-free fraction (lane 2), insoluble cell-free fraction (lane 3) and cell wall fraction (lane 4); from *S. venezuelae* pIJ86ΩC-His_6_-*vanY*_*n*_: extracellular fraction (lane 5), soluble cell-free fraction (lane 6), insoluble cell-free fraction (lane 7) and cell wall fraction (lane 8). A crude extract from *S. venezuelae* cells carrying the empty vector was loaded as a negative control (CTRL). In each lane, samples corresponding to 100 μL of cell culture were loaded. Std reference protein: C-His_6_-VanY_n_ from *E. coli* (5 μg, 25 kDa). The corresponding SDS-PAGE is shown in the Additional file 1: Figure S1. (**B**): quantitative analysis of recombinant VanY_n_ present in each fraction, performed using the Quantity One program (Bio-Rad Laboratories, Milan, Italy) and His_6_-VanY_n_ as comparative standard. Total protein content in each fraction was determined by Biuret method (□, solid line).

Cellular localization of His_6_-VanY_n_ was analyzed after 72 hours of growth in *S. venezuelae, S. coelicolor ΔvanRS* and *S. lividans* recombinant strains by Western blot analysis on different fractions (prepared as described in the Methods section). Figure 2A shows a ~ 25 kDa band corresponding to His_6_-VanY_n_ (predicted molecular mass of native VanY_n_ is 22.1 kDa) in the insoluble and soluble cell-free fractions and in the cell wall fractions from *S. venezuelae* recombinant strains grown in YEME medium. Recombinant VanY_n_ was never found in the concentrated broths (extracellular fractions, lanes 1 and 5 of Figure 2A). Densitometric analysis demonstrated that His_6_-VanY_n_ preferentially accumulated (90%, lanes 4 and 8 of Figure 2B) in the cell wall fractions obtained by the step of spheroplast preparation, independently on the localization of the His_6_-tag (at the C-terminus or at the N-terminus of the protein). Only 6% and 4% of recombinant protein was detected in insoluble and soluble cell-free fractions following spheroplast burst, respectively. For all recombinant strains grown in BTSB or YEME (Table 1), the heterologous protein distribution was exactly as for *S. venezuelae*, with most of VanY_n_ recovered from the cell wall fraction (data not shown).

### VanY_n_ production by *S. venezuelae* in 3 L bench-bioreactor scale

**Figure 3:**
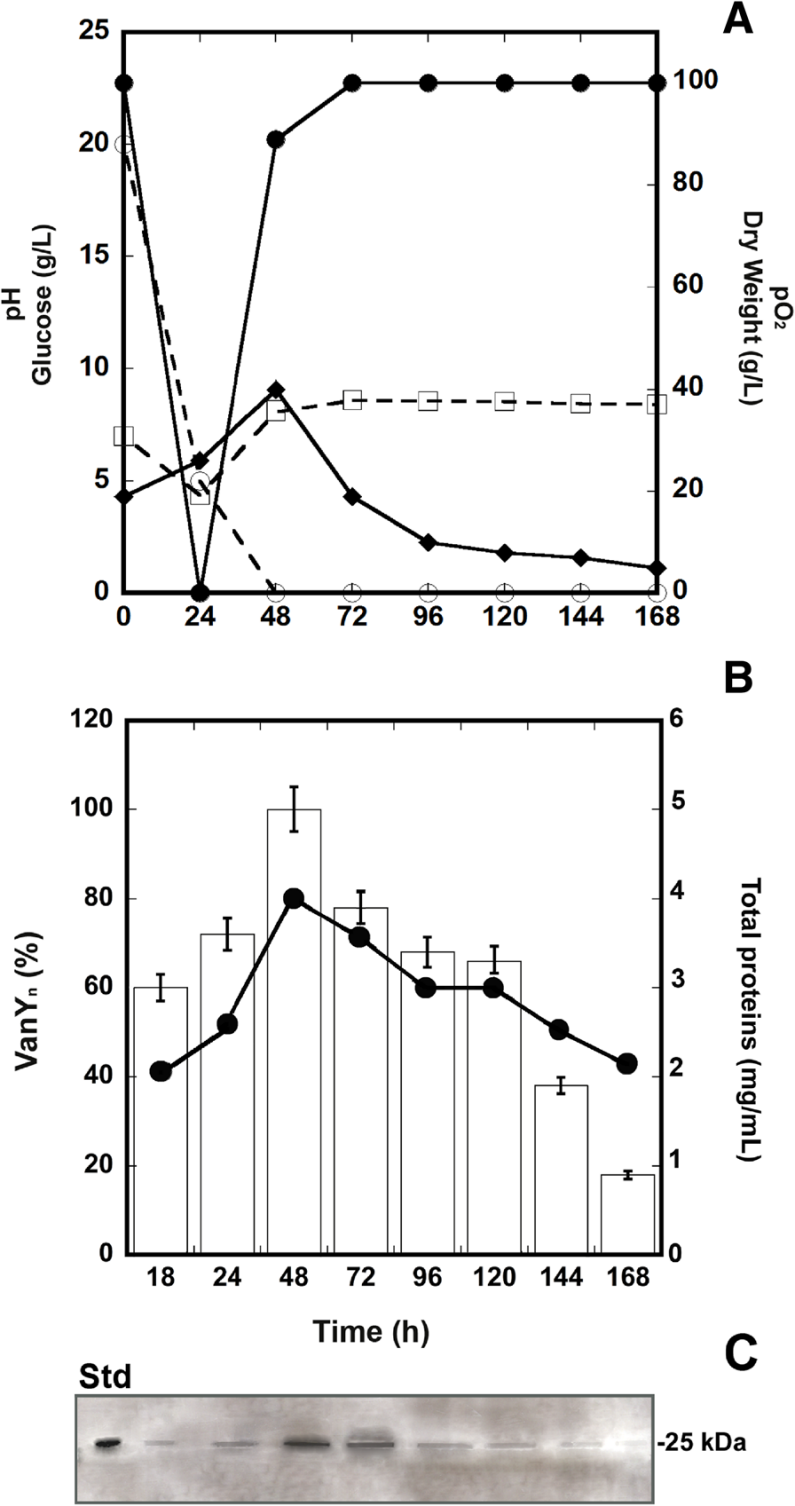
**Growth curve and VanY**_**n **_**production in 3-L batch fermentations of *S. venezuelae *pIJ86ΩC-His**_**6**_**-*vanY***_***n***_**in YEME.** (**A**) Time courses of pH (□, dashed line), pO_2_ (●, solid line), glucose (○, dashed line), and growth curve measured as dry weight (♦, solid line). (**B**) Time course of recombinant VanY_n_ production as determined by quantitative Western blot analysis of crude extracts from cell samples collected at different times of fermentation (see panel **C**). Maximum protein expression at 48 hours of growth was set as 100% (corresponding to ~ 30 mg/L of culture). Total protein content in each fermentation sample was determined by Biuret method (● , solid line). In (**C**), samples corresponding to 100 μL of cell culture were loaded in each lane; Std, standard reference protein: C-His_6_-VanY_n_ from *E. coli* (5 μg, 25 kDa). The corresponding SDS-PAGE is shown in the Additional file 2: Figure S2.

Both the forms of recombinant His_6_-VanY_n_ were produced growing *S. venezuelae* recombinant strains in YEME medium at 3 L bench-bioreactor scale. Figures 3A, B and C show the time course of growth and VanY_n_ production of *S. venezuelae* carrying pIJ86ΩC-His_6_*-vanY*_*n*_. Exponential growth was actually completed within 48 hours and gave a maximum biomass yield of 40 g/L dry weight. This growth phase was accompanied by a complete depletion of glucose from the medium and by a transient reduction of dissolved oxygen (pO_2_). Medium pH slightly decreased during the exponential growth phase and then tended to increase during the stationary growth phase. An estimated maximum of production of ~ 30 mg VanY_n_/L in the crude extract was determined at the peak of biomass production, i.e. at 48 hours from inoculum. An overlapping profile of growth curve and VanY_n_ production was similarly obtained for *S. venezuelae* carrying pIJ86ΩN-His_6_*-vanY*_*n*_ (not shown).

### VanY_n_ purification from *S. venezuelae*

**Figure 4:**
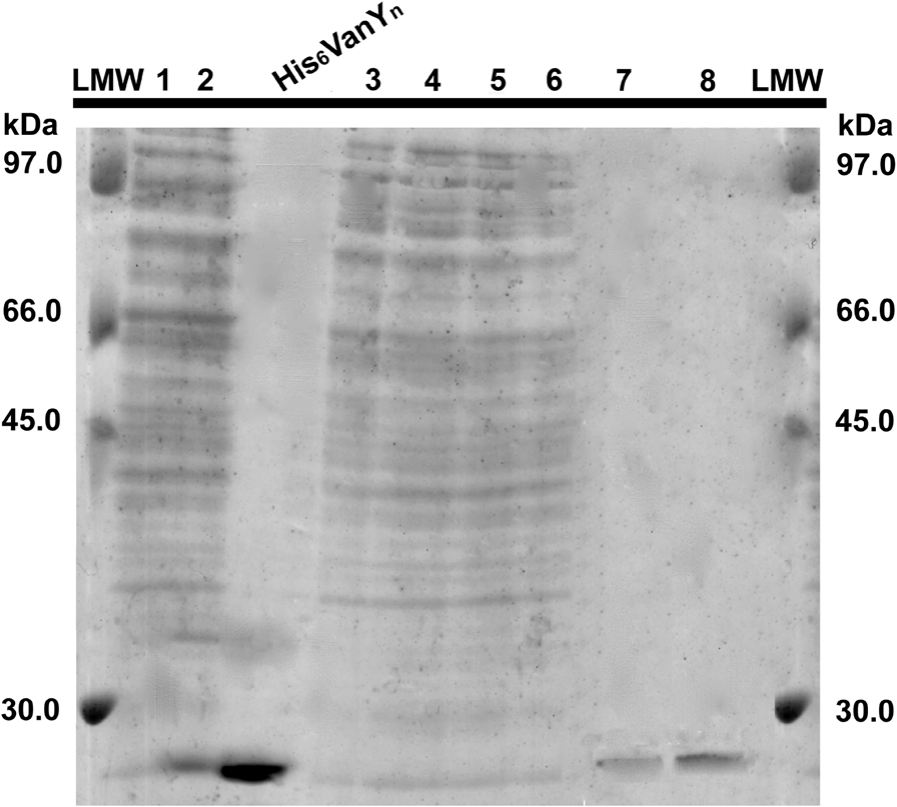
**Purification of recombinant VanY**_**n**_**.** SDS-PAGE Analysis (staining with Coomassie brilliant blue) of protein samples collected during purification of VanY_n_ from *S. venezuelae* carrying pIJ86ΩN-His_6_-*vanY*_*n*_ or pIJ86ΩC-His_6_-*vanY*_*n*_. Crude fractions from *S. venezuelae* carrying pIJ86ΩN-His_6_-*vanY*_*n*_ (lane 1) and *S. venezuelae* carrying pIJ86ΩC-His_6_-*vanY*_*n*_ (lane 2). Fractions from HiTrap Chelating chromatography: N-His_6_-VanY_n_ flow through (lane 3), C-His_6_-VanY_n_ flow through at 6 M (lane 4), 4 M (lane 5) and 1 M (lane 6) urea. Purified N-His_6_-VanY_n_ (lane 7, 2 μg) and C-His_6_-VanY_n_ (lane 8, 4 μg) after desalting gel permeation. The purified VanY_n_ migrated as a single band at a molecular mass of ~ 25 kDa and with > 90% purity. Standard reference protein: C-His_6_-VanY_n_ from *E. coli* BL21(DE3) Star (5 μg, 25 kDa). LMW, molecular mass standard proteins (Amersham, GE-Healthcare).

Purification of both the recombinant forms of His_6_-VanY_n_ from *S. venezuelae* cells grown in YEME medium, was attempted by means of metal-chelating chromatography on HiTrap chelating column of crude extracts obtained by whole cell sonication, as described in the Methods section. N-His_6_-VanY_n_ was eluted following standard procedures. In the same conditions, C-His_6_-VanY_n_ did not bind to the column, suggesting that the six histidines at C-terminus were masked. Accordingly, a denaturing agent such as urea was added to both the loading and equilibration buffers. The partially denatured C-His_6_-VanY_n_ protein interacted with the matrix and was subsequently re-folded directly on the column by a linear gradient of decreasing urea concentration. SDS-PAGE analysis (Figure 4) confirmed that both the tagged forms of His_6_-VanY_n_ migrated as a single band of 25 kDa and were > 90% pure. Final purification yield was comparable between the two forms: approximately 12 mg of C- or N-His_6_-VanY_n_ protein from one liter of culture were recovered.

### Enzymatic activity of pure recombinant VanY_n_

Activities of purified C-His_6_-VanY_n_ and N-His_6_-VanY_n_ from *S. venezuelae* recombinant strains were assayed on commercially available surrogates of peptidoglycan precursors, in parallel with the previously characterized C-His_6_-VanY_n_ produced by recombinant *E. coli*[17]. These results were obtained by the d-amino acid oxidase/peroxidase colorimetric coupled reaction [18,19], and confirmed by a fluorimetric assay [20]. As shown in Table 2, C-His_6_-VanY_n_ from *S. venezuelae* cleaved the last d-Ala from the tripeptide N_α_,N_ε_-diacetyl-l-Lys-d-Ala-d-Ala and acetyl-l-Lys-d-Ala-d-Ala, this activity being only slightly affected by the acetylation grade of the Lys. The activity was halved if the substrate was the d-Ala-d-Ala. Thus, C-His_6_-VanY_n_ showed a higher d,d-carboxypeptidase activity (VanY-like) than a d,d-peptidase activity (VanX-like). Surprisingly, N-His_6_-VanY_n_ from *S. venezuelae* did not show any activity on the three substrates (Table 2), suggesting that the tag position dramatically influenced enzyme competence.

**Table 2:** **Substrate specificity of His**_**6**_**-VanY**_**n **_**recombinant forms produced by different hosts**

	***S. venezuelae***		***E. coli***
**Substrate**	**C-His**_**6**_**-VanY**_**n**__**(U/mg)**_	**N-His**_**6−**_**VanY**_**n**__**(U/mg)**_	**C-His**_**6−**_**VanY**_**n (U/mg)**_
d-Ala-d-Ala	18 ± 5.0	0	19 ± 3
N_α_,N_ε_-diacetyl-l-Lys-d-Ala-d-Ala	38 ± 3.8	0	36 ± 4.0
Acetyl-l-Lys-d-Ala-d-Ala	40 ± 5.6	0	40 ± 3.0

Specific activity (U/mg protein) was determined using 40 μg of His_6_-VanY_n_ added to 10 mM solutions of the indicated compounds. The activity was assayed at 25°C as described in the Methods section. Results are the average of three independent experiments.

**Figure 5:**
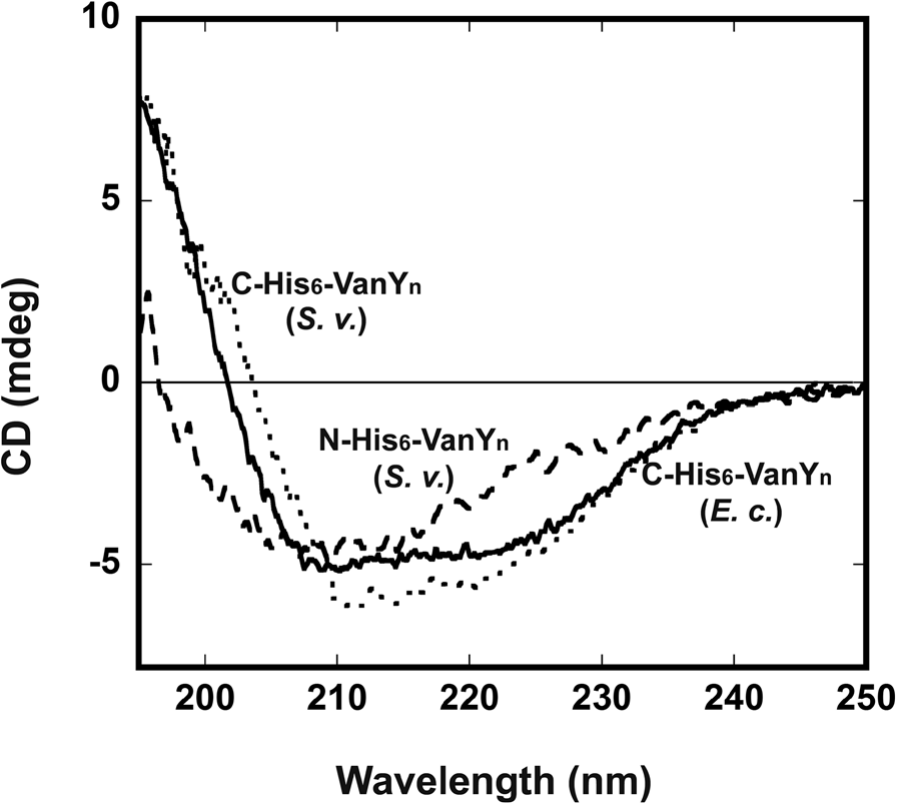
**Far-UV CD spectra of recombinant VanY**_**n**_**.** C-His_6_-VanY_n_ (dotted line) and N-His_6_-VanY_n_ (dashed line) from recombinant *S. venezuelae* (*S. v.*); C-His_6_-VanY_n_ from recombinant *E. coli* (*E. c.*) (continuous line). Protein concentration was 0.1 mg/mL, 15°C.

These results were supported by the circular dichroism (CD) spectra of the recombinant proteins. CD spectrum of C-His_6_-VanY_n_ produced in *S*. *venezuelae* overlapped with that of the protein produced in *E. coli* (Figure 5): analysis of secondary structure indicates a predominance of β-sheets (~ 38%) and ~ 15% of α-helices. This structure content was altered for the N-His_6_-VanY_n_ (Figure 5), indicating that N-terminal tag interfered with the proper protein folding and secondary structure content of the recombinant enzyme.

### Resistance phenotype in *S. venezuelae* recombinant strains

Role of different constructs of *vanY*_*n*_, when over-expressed in a glycopeptide-susceptible heterologous host such as *S. venezuelae*, was investigated *in vivo* by determining the d,d-peptidase/d,d-carboxypeptidase activity of alkaline cell extracts and the glycopeptide resistance phenotype of recombinant strains. As shown in Table 3, d,d-peptidase/d,d-carboxypeptidase activity was detectable only in *S. venezuelae* pIJ86ΩC-His_6_-*vanY*_*n*_ and *S. venezuelae* pIJ86Ω*vanY*_*n*_ (a strain with *vanY*_*n*_ cloned without any tag, see [12]), but not in *S. venezuelae* pIJ86ΩN-His_6_-*vanY*_*n*_ neither in *S. venezualae* wild-type or in *S. venezualae* containing the empty vector. Table 3 also reports the minimal inhibitory concentrations (MICs) of vancomycin and teicoplanin to the recombinant strains in comparison with the wild-type. Accordingly to the expression of the d,d-peptidase/d,d-carboxypeptidase activity, *S. venezuelae* expressing C-His_6_-VanY_n_ and *S. venezuelae* producing VanY_n_ without any tag showed an increased resistance level against both glycopeptides. Resistance phenotype of *S. venezuelae* producing the N-His_6_-VanY_n_ was the same as in the control strain with empty vector or in the wild-type.

**Table 3:** **Resistance and enzyme activity profiles of *S. venezuelae *strains**

	**MIC**		**Enzyme activity**	
**Strain**	**Vancomycin (μg/mL)**	**Teicoplanin (μg/mL)**	**d,d**-**carboxypeptidase (U/mg)**	**d,d**-**peptidase (U/mg)**
wild-type	0.2	0.5	0	0
pIJ86	0.2	0.5	0	0
pIJ86ΩC-His_6_-*vanY*_*n*_	0.4	0.7	189 ± 5.5	100 ± 3.9
pIJ86ΩN-His_6_-*vanY*_*n*_	0.2	0.5	0	0
pIJ86Ω*vanY*_*n*_	0.4	0.7	172 ± 4.3	80 ± 3.2

MICs of glycopeptides were determined by plating 10^6^ cfu/mL of each strain on agar medium added with different antibiotic concentrations. Results are the average of three independent experiments in which the standard deviation was less than 5%. Specific activity (U/mg protein) was determined in alkaline cell extracts added to 10 mM solutions of acetyl-l-Lys-d-Ala-d-Ala or d-Ala-d-Ala. The activity was assayed at 25°C as described in the Methods section [17]. Results are the average of three independent experiments.

These data confirm the role of VanY_n_ in conferring glycopeptide resistance to a susceptible host and indicate that C-terminal His_6_-VanY_n_ behaved *in vivo* as the native VanY_n_, whereas the addition of His_6_ at the N-terminus of the protein abolished its biological activity*.*

## Discussion

C-His_6_-VanY_n_ was previously produced in BL21(DE3) Star *E. coli* cells as a cytoplasmatic soluble protein [13]. In that case, codon usage optimization of the synthetic gene was essential since *E. coli* is a low G-C ratio (~ 50%) Gram-negative bacterium whereas *Nonomuraea* sp. is a high G-C Gram-positive actinomycete. The G-C ratio of *vanY*_*n*_ is 73.3% which is similar to the genomic ratio of the reference *S. coelicolor* A3(2) (72%) and *S. venezuelae* ETH14630 (71%) [14]. The G-C ratio of the *dbv* cluster is 70.4%. Notwithstanding our efforts in optimizing cultivation, induction and purification conditions, the best productivity achieved in recombinant *E. coli* was 4.6 mg/L culture (0.13 mg/g cells) [17]. Similar level of expression was obtained by other authors who cloned *vanXY*_*C*_ from *Enterococcus gallinarum* BM4174 in *E. coli* JM83 [21], *vanX* from *Enterococcus faecium* BM4147 in *E. coli* W3110 [22] and *vanY* from *Enterococcus faecium* BM4147 in *E. coli* JM83 [20]. VanXY_C_, VanX and VanY are d,d-carboxypeptidases/d,d-peptidases homologous to VanY_n_ involved in conferring glycopeptide resistance to enterococci, which are Gram-positive pathogens with low G-C content [23,24].

By cloning *vanY*_*n*_ from *Nonomuraea* sp. ATCC 39727 in the taxonomically closely related *S. venezuelae*, pure protein production was at least three folds higher (12 mg/L culture) than in *E. coli*. Since estimated volumetric productivity in the harvested cells was around 30 mg of protein per liter of culture, there is still room to improve the final yield by further optimization of the purification procedure. Estimated specific productivity of around 1 mg VanY_n_ per gram of cells in streptomycetes was much higher than the one achieved in *E. coli* (0.13 mg/g cells) confirming that streptomycetes are preferable hosts for VanY_n_ production. Comparable biomass and protein production were achieved at flask and at 3 L bioreactor-scale: maximum values being one day anticipated in bioreactor runs. Fermentation and downstream technologies are well developed for streptomycetes, which are industrially relevant microorganisms producing many valuable antibiotics and other bioactive metabolites [1]; thus they can be successfully further applied to improving and scaling-up VanY_n_ production.

An important difference between heterologous production of VanY_n_ in recombinant streptomycetes and *E. coli* is the preferential subcellular localization of the protein. VanY_n_ (196 amino acids) contains three different putative domains: a cytoplasmatic domain at the N-terminus (the first 20 amino acids), an hydrophobic trans-membrane portion (20 amino acids), followed by the C-terminal domain exposed on the external face of cytoplasmatic membrane. This extracellular domain contains conserved motifs (SxHxxGxAxD and ExxH) for the coordination of zinc ions and the catalytic active site [23,25,26]. In *E. coli* recombinant cells, C-His_6_-tagged*-*VanY_n_ was accumulated in the cytoplasm, whereas in streptomycetes the most (90%) of the tagged enzyme was recovered from the cell wall fraction. The localization of VanY_n_ confirms its role in the extracellular phases of cell wall biosynthesis [23,25]. The addition of detergents did not improve purification from the whole cell extract, confirming that the protein is easily detached from membranes. VanY d,d-carboxypeptidases from glycopeptide resistant enterococci are in fact involved in the extracytoplasmatic hydrolysis of the last d-Ala from the UDP-pentapeptide PG precursors [20].

The enzymatic activity and the spectral investigation (by CD spectroscopy) confirmed the identity of C-His_6_-VanY_n_ produced by recombinant cells of either *S. venezuelae* or *E. coli.* On substrates that mimic peptidoglycan precursors, VanY_n_ showed d,d-carboxypeptidase and d,d-dipeptidase activity, resembling more VanXY_C_ from VanC-type *Enterococcus gallinarum*[21] than typical VanY d,d-carboxypeptidases and VanX d,d-peptidases firstly characterized in *Enterococcus faecium* BM4147 [20,27,28]. On the other hand, the introduction of an histidine hexamer at the N-terminus of VanY_n_ abolishes the enzymatic activity and alters protein secondary structure, suggesting that it interferes with the folding of the active protein. Till now, VanY family of d,d-carboxypeptidases have not been structurally investigated. The possibility to produce discrete amounts of pure VanY_n_ by using *S. venezuelae* as a cell factory open the way to better investigate the peculiar bifunctional activity of this d,d-dipeptidase/d,d-carboxypeptidase and its interaction with substrates and inhibitors.

Finally, the functional study of recombinant VanY_n_ activity in cellular extracts confirmed the role of this protein in conferring glycopeptide resistance in a susceptible host such as *S. venezuelae*, which lacks those *vanRSHAX* genes commonly considered essential to confer glycopeptide resistance [24]. Our data show that when *vanY*_*n*_ gene was produced in the biologically active form (C-His_6_-VanY_n_ or VanY_n_ without any tag), its expression conferred resistance in the absence of *vanRSHAX* genes, albeit at a reduced level, as previously demonstrated in the homologous producer *Nonomuraea* sp. ATCC 39727 [12]. As proof of this, expression of the inactive N-His_6_-VanY_n_ form did not increase the level of resistance towards two glycopeptides (vancomycin and teicoplanin) of the recombinant hosts, which remains the same as in the wild-type and as in the control recombinant strain containing only the empty vector.

## Conclusions

*Streptomyces* spp. were demonstrated valuable hosts for the production of specific peptidases, involved in cell wall turnover of glycopeptide resistant microbial cells. The novel d,d-dipeptidase/d,d-carboxypeptidase VanY_n_ from the glycopeptide producer *Nonomuraea* sp. ATCC 39727 was successfully produced in a good yield and in the biologically active form conferring resistance to the glycopeptide susceptible *S. venezuelae* strain. Developing such streptomyces cell factory system for VanY_n_ production opens the way to a further characterization of the enzyme, to a better comprehension of its role in glycopeptide resistance, and to its use as novel biocatalyst.

## Methods

### Strains and media

*Nonomuraea* sp. ATCC 39727 was maintained and cultivated to prepare genomic DNA according to [12]. *Streptomyces coelicolor ΔvanRS* was gently donated by Hee-Jeon Hong, University of Cambridge, UK [16], *Streptomyces lividans* TK24 and *Streptomyces venezuelae* ATCC 10595 were a gift from Mervyn Bibb, John Innes Institute, Norwich, UK [14]. *Streptomyces* spp. strains were maintained as spores in 10% (v/v) glycerol and propagated in MYM and SFM agar media [14]. For growing ex-conjugants containing pIJ86, pIJ86ΩC-His_6_-*vanY*_*n*_, pIJ86ΩN*-vanY*_*n*_ and pIJ86Ω*vanY*_*n*_, agar plates were added with 50 μg/mL of apramycin (Sigma-Aldrich, Milan, Italy). Agar plates were incubated at 28°C. Liquid media for streptomycetes were YEME -containing in (w/v) 0.3% yeast extract, 0.5% bacto-peptone, 0.3% oxoid malt extract, 1% glucose in deionized water, pH 7.0- and BTSB -containing in (w/v) 10% sucrose, 1% yeast extract, 1% glucose, 0.5% NaCl, 0.5% soybean meal, 1.7% tryptone and 0.25% K_2_HPO_4_ in deionized water, pH 7.0. All medium components were from Sigma-Aldrich (Milan, Italy), unless otherwise stated. Colonies were picked up from agar plates and inoculated into 300 mL baffled flasks containing 50 mL of YEME or BTSB. Flask cultures were incubated on a rotary shaker at 200 rpm and 28°C. Media and culture conditions for *E. coli* were described in [12]. *E. coli* DH5α was used as host for plasmid construction. *Escherichia coli* ET12567/pUZ8002 [5] was used as non-methylating plasmid donor strain for intergeneric conjugation with *Streptomyces* spp. Cells were propagated in Luria-Bertani (LB) (Sigma-Aldrich, Milan, Italy) broth at 37°C.

### Plasmids

pIJ86 (gift from Mervyn Bibb) was used as a multi-copy vector for heterologous expression in *Streptomyces* spp. [12]. Plasmids pIJ86ΩC-His_6_-*vanY*_*n*_ and pIJ86ΩN-His_6_-*vanY*_*n*_ were constructed as follow. Expand High Fidelity polymerase (Roche, Milan, Italy) was used to amplify *vanY*_*n*_ using genomic DNA of *Nonomuraea* sp. ATCC 39727 as template with oligonucleotide primers *vanY*004*Fw* (5^′^-ATA**GGATCC**CCAGACTGGAGGAGAGGGATGAGGAGAAGCGAGGGTGAC-3^′^) and *vanY*004*Rev* (5^′^-GAT**AAGCTT**CTAGTGGTGGTGGTGGTGGTGGACCCGGCCCCCGTTCCGGCT-3^′^) that introduced a C-terminal tag of six histidine residues and the *HindIII* and *BamHI* (Roche, Milan, Italy) restriction sites, respectively, into the PCR product, allowing insertion into the multiple cloning site of the multicopy expression vector pIJ86. The *vanY*_*n*_ with a N-terminal tag of six histidines was produced using as oligonucleotide primers *vanY*003*Fw* (5^′^-ATATTT**GGATCC**ATGCACCACCACCACCACCACAGGAGAAGCGAGGGTGACGAC-3^′^) and *vanY*003*Rev* (5^′^-GAT**AAGCTT**CCCGTGCCCTAGCTAGACCCGGCCCCCGTTCCGGCT-3^′^) that introduced *HindIII* and *BamHI* restriction sites. The *vanY*_*n*_ without any tag was produced using as oligonucleotide primers *vanY86Fw* (5^′^-AT**GGATCC**CAGACTGGAGGAGAGGGATG-3^′^) and *vanY86Rev* (5^′^-GAT**AAGCTT**CGATCCTGGAGTTCGTCTTC-3^′^) that introduced *BamHI* and *HindIII* restriction sites [12]. The PCR products were purified, digested with *HindIII* and *BamHI*, and ligated with pIJ86 vector that had similarly been digested, to produce pIJ86ΩC-His_6_*-vanY*_*n*_, pIJ86ΩN-His_6_*-vanY*_*n*_ and pIJ86Ω*vanY*_*n*_. These vectors with *vanY*_*n*_ transcribed from the strong constitutive ermE* promoter [14] were used to transform *E. coli* ET12567/pUZ8002 cells.

### Intergeneric conjugation

Intergeneric conjugation was performed according to a modified protocol from [5,14]. In brief, a culture of the donor *E. coli* ET12567/pUZ8002 containing the selected plasmid was grown in 10 mL LB supplemented with 50 μg/mL apramycin, 25 μg/mL chloramphenicol (Sigma-Aldrich, Milan, Italy) and 50 μg/mL kanamycin (Sigma-Aldrich, Milan, Italy) to an OD_600nm_ of 0.4. Cells were collected by centrifugation, washed twice with an equal volume of LB, and resuspended in 1 mL of LB. For each conjugation approximately 10^8^*Streptomyces* spp. spores, collected from agar plates in sterile glycerol, were added to 500 μL 2X YT broth [14], heat shocked at 50°C for 10 minutes and then allowed to cool. 500 μL of *E. coli* cells were added to 500 μL of heat-shocked spores and mixed briefly. Mixture was plated out on SFM or MYM agar added with 10 mM MgCl_2_ and incubated for 20 hours at 30°C. Plates were overlaid with 1 mL of water containing 500 μg/mL nalidixic acid (Sigma-Aldrich, Milan, Italy) and 50 μg/mL apramycin and were incubated at 30°C until colonies appear.

### Colony PCR and sequencing

The presence of pIJ86Ω*vanY*_*n*_, pIJ86ΩC-His_6_-*vanY*_*n*_ or pIJ86ΩN-His_6_-*vanY*_*n*_ was checked by colony PCR and DNA sequencing of ex-conjugants. Single colonies were transferred onto DNA medium [14]. Plates were incubated at 30°C overnight. Mycelium was scraped from the plates using a sterile toothpick and was introduced into 50 μL 100% v/v DMSO in a 1.5 mL tube. The tube was shaken vigorously for 1–2 hours and then centrifuged: 2.5 μL of the supernatant were used for PCR. For control, 1 μL of DNA samples were mixed with 1.5 μL DMSO. One initial step of 10 minutes at 95°C was included in the PCR program to ensure the complete cell lysis. The PCR was performed for 30 cycles as follows: 95°C for 45 seconds, 56°C for 30 seconds and 72°C for 1 minute. *vanY*004*Fw*, *vanY*004*Rev*, *vanY*003*Fw* and *vanY*003*Rev, vanY86Fw* and *vanY86Rev* were used as oligonucleotide primers.

### VanY_n_ expression

Recombinant *Streptomyces* spp. were grown aerobically in 100 mL YEME and BTSB media added with apramycin (50 μg/mL) in 500 mL Erlenmayer flasks for different time intervals (up to 196 h) at 28°C and 200 rpm. Cells were collected by centrifugation at 39000 × g for 15 minutes and washed three times with water. The supernatant (named “extracellular fraction”) was collected and precipitated with the trichloroacetic acid (TCA) method. TCA precipitation was performed adding 1/10 (v/v) of 100% TCA (w/v) to an appropriate medium volume and vortexed for 15 seconds, placed on ice for 15 minutes and then centrifugated at 14,000 × *g* for 10 minutes. The supernatant was removed and discarded. The pellet was washed twice with 100 μL of pure acetone and then air dried for about 60 minutes.

Washed cells were suspended in 10 mL of 0.1 M Tris–HCl buffer (pH 7.6) containing 0.4 M sucrose and 6.7 mg of lysozyme (Sigma-Aldrich, Milan, Italy). After 6 hours of incubation at 37°C, the mixture was centrifuged at 12800 × *g* for 10 minutes, obtaining spheroplasts clearly distinguishable at the optical microscopic observation (40X, Zeiss Primo Star microscope, Arese, Italy). The supernatant thus obtained was named “cell wall fraction”. The spheroplasts were washed with 0.4 M sucrose and then 5 mL of water was added to burst the spheroplasts. The suspension was centrifuged at 2000 × *g* for 30 minutes. The supernatant and the precipitate were named as “cell-free soluble extract” and “cell-free insoluble fraction”, respectively.

### Scale up in 3-L reactor

Flask cultures of recombinant *Streptomyces* spp. grown in YEME and apramycin (50 μg/mL) for 48–72 hours were used to inoculate - at 2.5% (v/v) - 3-L P-100 Applikon glass reactor (height 25 cm, diameter 13 cm) equipped with a AD1030 Biocontroller and AD1032 motor, containing 2 L YEME and apramycin (50 μg/mL). Cultivations in fermenter were carried out for 168 hours at 28°C, with stirring at 500 rpm (corresponding to 1.17 m/s of tip speed) and 2 L/min aeration rate. Dissolved oxygen (measured as % of the initial pO_2_ value) was monitored using an Ingold polarographic oxygen electrode. The pH values of culture broths were monitored using a pH meter. Foam production was controlled by adding Antifoam SE-15 (Sigma-Aldrich, Milan, Italy) through an antifoam sensor.

### VanY_n_ purification

For protein purification, cultures of recombinant *S. venezuelae* grown in YEME medium were harvested and centrifuged at 39000 × *g* for 15 min at 4°C. Cells were sonicated in a buffer solution 300 mM NaCl, 30 mM imidazole, 10 μg/mL DNAseI (Sigma-Aldrich, Milan, Italy), 0.19 mg/mL PMSF (Sigma-Aldrich, Milan, Italy) and 0.7 μg/mL pepstatine (Sigma-Aldrich, Milan, Italy) in 50 mM potassium phosphate buffer (pH 7.0), for 15 cycles of 30 seconds each on ice. After a centrifugation at 39000 × *g* for 60 min at 4°C, the supernatant was collected. N-His_6_-VanY_n_ was purified by affinity chromatography onto a HiTrap chelating affinity column (1.6 × 2.5 cm, 5 mL, GE Healthcare Sciences, Milan, Italy) equilibrated with 50 mM potassium phosphate buffer (pH 7.0) containing 30 mM imidazole and 300 mM NaCl, according to the manufacturer’s instructions. After extensive washing, the bound protein was eluted with 50 mM potassium phosphate buffer (pH 7.0) containing 300 mM NaCl and 300 mM imidazole [17]. For C-His_6_-tagged VanY_n_, 6 M urea was added to the equilibration buffer as denaturant; in order to refold the protein bound to the column, a linear gradient from 6 to 0 M of urea (2 mL/min) was performed before starting the elution phase [29]. Fractions containing pure recombinant VanY_n_ were loaded on PD10 Sephadex G25 column (Ge Healthcare Sciences, Milan, Italy) equilibrated with 50 mM potassium phosphate buffer (pH 7.0). Protein purity was checked by SDS-PAGE (using 15% polyacrylamide gels and staining with Coomassie brilliant blue) and Western blot analysis. Protein concentration was estimated using the extinction coefficient at 280 nm (45258 M^-1^ cm^-1^) determined by urea denaturation and the theoretical extinction coefficient based on amino acid sequence.

### Western blot analysis

Following electrophoresis of proteins from bacterial cell fractions (corresponding to 100 μL of culture) or affinity-purified fractions and the transfer to a nitrocellulose sheet (GE Healthcare Sciences, Milan, Italy), the membrane was incubated with 1:1000 (v/v) His•Tag® Antibody HRP Conjugate Kit (Novagen, Milan, Italy) in alkalin-casein solution 1% (v/v). The immunorecognition was visualized by ECL Detection Reagents (GE Healthcare Sciences, Milan, Italy). The quantitative analysis was performed using the bioinformatics program Quantity One (Bio-Rad Laboratories, Milan, Italy) and C-His_6_-VanY_n_ from *E. coli* as standard protein. For molecular mass determination, PageRuler™ Prestained Protein Ladder (Thermo Scientific, Milan, Italy) markers were used. Protein content in each fraction was assayed by Biuret method and SDS-PAGE analysis (Additional file 1: Figure S1 and Additional file 2: Figure S2).

### Alkaline extraction of d,d-carboxypeptidase

All manipulations were carried out at 0 to 4°C. Cells at different growth phases were harvested and suspended in 2 ml per gram of cells of physiological solution (0.9% (v/v) NaCl). The mycelium was fragmented by mild sonication and cells were collected by centrifugation at 39,000 × *g* for 15 minutes. Alkaline extractions were carried out by suspending the cell suspension in ice-cold distilled water containing the proteinase inhibitors (0.19 mg/ml PMSF (Sigma-Aldrich, Milan Italy) and 0.7 μg/ml pepstatine (Sigma-Aldrich, Milan Italy) and then bringing the suspension to pH 12 by adding an appropriate volume of 2.5 N NaOH. After centrifugation (28,000 × *g*, 15 min, 4°C), the supernatants were neutralized to pH 7 by the addition of 0.5 M sodium acetate (pH 5.4) [30].

### d,d-dipeptidase and d,d-carboxypeptidase assays

Enzyme activity was assayed by measuring the release of d-Ala from N_α_,N_ε_-diacetyl-l-Lys-d-Ala-d-Ala, acetyl-l-Lys-d-Ala-d-Ala and d-Ala-d-Ala by a d-amino acid oxidase/peroxidase coupled colorimetric assay (i) or by reaction with a fluorescent reagent (ii). All substrates were purchased from Sigma-Aldrich. One unit of d,d-carboxypeptidase activity was defined as the amount of enzyme that produced 1 μmol of d-Ala per min.

(i) D-Amino acid oxidase/peroxidase assay [17,18]. Reaction mixtures contained 10 mM of the substrate (N_α_,N_ε_-diacetyl-l-Lys-d-Ala-d-Ala, or acetyl-l-Lys-d-Ala-d-Ala or d-Ala-d-Ala), 5 mM of the peroxidase colorimetric substrate 4-aminoantipyrine (4-AAP, from Sigma-Aldrich, Milan, Italy), 3 U/mL *Rg*DAAO (d-amino acid oxidase from *Rhodotorula gracilis*[18]), 7.5 U/mL horseradish peroxidase (HRP from Sigma-Aldrich, Milan, Italy), 6 mM phenol in 50 mM 1,3-bis[tris(hydroxymethyl)methylamino]propane (pH 7.4) in a final volume of 1 mL. At 25°C, 40 μg of recombinant VanY_n_ or the amount of cell wall alkaline extract correspondent to 50 mg of cells, or the soluble fraction or the supernatant, was added to the reaction mixture and the increase in absorbance (ΔAbs/min) at 510 nm was measured for the test sample as well as for the control to which no VanY_n_ was added. A molar extinction coefficient for chinonemine of 6.58/mM cm was used.

(ii) Fluorimetric *o*–phthaldialdehyde (OPTA) method [20]. Reaction mixtures contained 10 mM N_α_,N_ε_-diacetyl-l-Lys-d-Ala-d-Ala, 40 μg of recombinant VanY_n_ in 50 mM phosphate buffer (pH 7.0) in a final volume of 200 μL. After 10 minutes at 25°C, the reaction was stopped by adding 50 μL of 250 mM HCl followed by 750 μL of water. Enzymatically released d-Ala was detected by the addition of 100 μL of fluoraldehyde (OPTA) solution (Sigma-Aldrich, Milan, Italy) to 100 μL of the reaction mix, followed by incubation at room temperature for 5 min. 800 μL of water was added and 200 μL was removed: the fluorescence intensity was measured (λ_ex_ = 340 nm; λ_em_ = 455 nm) in a fluorescence microplate reader (Tecan Infinite® 200 Pro, Milan, Italy). Assays were quantified from a standard curve prepared with known amounts of d-Ala.

### Circular dichroism measurements

Far-UV CD spectra were recorded with a Jasco J-715 (Jasco Europe, Cremella, Italy) spectropolarimeter in the 195–250 nm wavelength range. Measurements were made in quartz cuvettes of 1 mm pathlength, employing protein solutions of 0.1 mg/mL, and were corrected for buffer contribution. Secondary structure fractions were calculated from deconvolution of the CD spectra using the program K2D2 (http://www.ogic.ca/projects/k2d2/) [31].

### Determination of the biological activity: the minimum inhibitory concentration (MIC)

Minimal inhibitory concentrations (MICs) of teicoplanin and vancomycin (Sigma-Aldrich, Milan, Italy) to *S. venezuelae* containing pIJ86 or pIJ86ΩC-His_6_-*vanY*_*n*_ or pIJ86ΩN-His_6_-*vanY*_*n*_ was determined in MYM agar added with 50 μg/mL apramycin and increasing concentrations of glycopeptides. The inoculum was 10^6^ cfu/mL (after mycelium sonication with Sonics Vibracell VCX 130 – power 130 Watt, 230 Volt, 50–60 Hertz, frequency 20 Hz 5 minutes of sonication, 20 seconds for each cycles with 90% of amplitude), and plates were incubated at 28°C until colonies appeared. MIC was the lowest concentration of the antibiotic that inhibits the visible growth of the recombinant *S. venezuelae* strains [32].

## Competing interests

The authors declare that they have no competing interests.

## Authors’ contributions

FM conceived the project and wrote the paper. EB performed most of the experiments on protein expression, purification and biochemical characterization, and prepared figures and tables. GLM developed conjugation and molecular biology tools. FB focused on protein purification procedures. LP designed the experiments on the protein biochemistry. All authors have read and approved the final manuscript.

## Supplementary Material

Additional file 1: Figure S1 SDS-PAGE Analysis of C-His_6_-VanY_n_ and N-His_6_-VanY_n_ from *S. venezuelae* recombinant strains as in Figure 2 main text. Analysis of samples corresponding to cellular and extracellular fractions from cultures of *S. venezuelae* recombinant strains grown in YEME for 72 hours. From *S. venezuelae* pIJ86ΩN-His_6_-*vanY*_*n*_: extracellular fraction (lane 1), soluble cell-free fraction (lane 2), insoluble cell-free fraction (lane 3) and cell wall fraction (lane 4); from *S. venezuelae* pIJ86ΩC-His_6_-*vanY*_*n*_: extracellular fraction (lane 5), soluble cell-free fraction (lane 6), insoluble cell-free fraction (lane 7) and cell wall fraction (lane 8). In each lane, samples corresponding to 100 μL of cell culture were loaded. Std reference protein: C-His_6_-VanY_n_ from *E. coli* (5 μg, 25 kDa). Click here for file

Additional file 2: Figure S2 SDS-PAGE Analysis of C-His_6_-VanY_n_ from *S. venezuelae* recombinant strain growth in 3-L batch fermentor as in Figure 3 main text. Crude extracts of cell samples collected at different times of fermentation: 18 (lane 1), 24 (lane 2), 48 (lane 3), 72 (lane 4), 96 (lane 5), 120 (lane 6), 144 (lane 7), 168 (lane 8) hours. In each lane, samples corresponding to 100 μL of cell culture were loaded. Std reference protein: C-His_6_-VanY_n_ from *E. coli* (5 μg, 25 kDa). Click here for file
