# Supplementary material for: Streptomyces spp. as efficient expression system for a d,d-peptidase/d,d-carboxypeptidase involved in glycopeptide antibiotic resistance
Source: BMC Biotechnol. 2013 Mar 16;13:24. doi: 10.1186/1472-6750-13-24 (PMC3610138; doi:10.1186/1472-6750-13-24)
Supplement: Additional file 1: Figure S1 — SDS-PAGE Analysis of C-His6-VanYn and N-His6-VanYn from S. venezuelae recombinant strains as in Figure 2 main text. Analysis of samples corresponding to cellular and extracellular fractions from cultures of S. venezuelae recombinant strains grown in YEME for 72 hours. From S. venezuelae pIJ86ΩN-His6-vanYn: extracellular fraction (lane 1), soluble cell-free fraction (lane 2), insoluble cell-free fraction (lane 3) and cell wall fraction (lane 4); from S. venezuelae pIJ86ΩC-His6-vanYn: extracellular fraction (lane 5), soluble cell-free fraction (lane 6), insoluble cell-free fraction (lane 7) and cell wall fraction (lane 8). In each lane, samples corresponding to 100 μL of cell culture were loaded. Std reference protein: C-His6-VanYn from E. coli (5 μg, 25 kDa). [file 1472-6750-13-24-S1.pdf]

Additional file 1

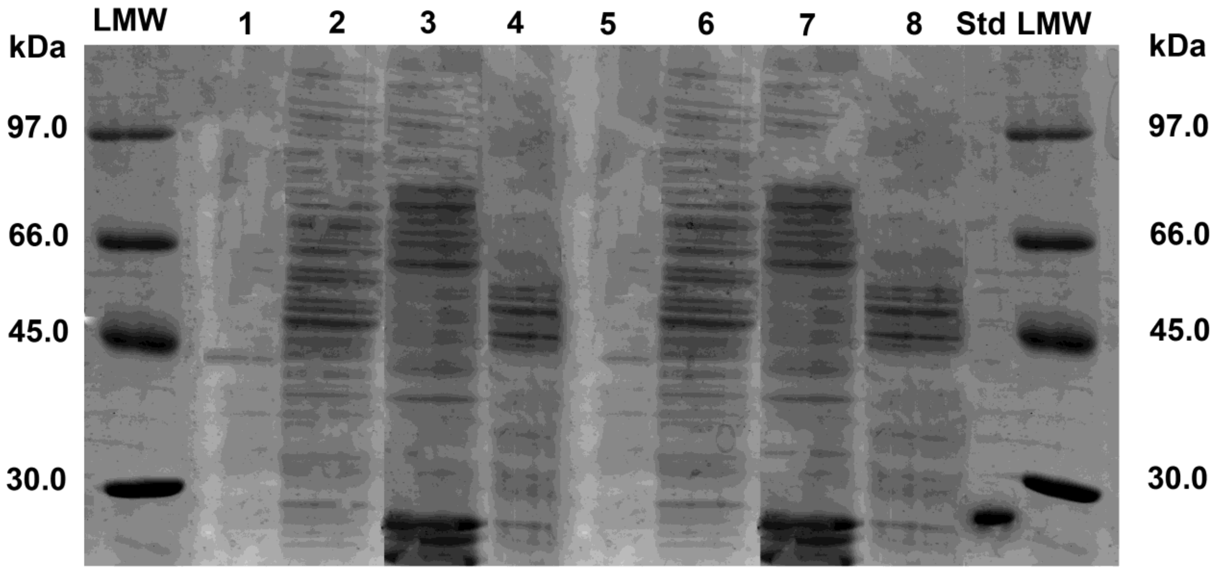

Additional Figure 1

SDS-PAGE Analysis of C-His$_6$-VanY$_n$ and N-His$_6$-VanY$_n$ from *S. venezuelae* recombinant strains as in Fig. 2 main text. Analysis of samples corresponding to cellular and extracellular fractions from cultures of *S. venezuelae* recombinant strains grown in YEME for 72 hours. From *S. venezuelae* pIJ86ΩN-His$_6$-*vanY$_n$*: extracellular fraction (lane 1), soluble cell-free fraction (lane 2), insoluble cell-free fraction (lane 3) and cell wall fraction (lane 4); from *S. venezuelae* pIJ86ΩC-His$_6$-*vanY$_n$*: extracellular fraction (lane 5), soluble cell-free fraction (lane 6), insoluble cell-free fraction (lane 7) and cell wall fraction (lane 8). In each lane, samples corresponding to 100 µL of cell culture were loaded. Std reference protein: His$_6$-VanY$_n$ from *E. coli* (5 µg, 25 kDa).
