# Supplementary material for: Streptomyces spp. as efficient expression system for a d,d-peptidase/d,d-carboxypeptidase involved in glycopeptide antibiotic resistance
Source: BMC Biotechnol. 2013 Mar 16;13:24. doi: 10.1186/1472-6750-13-24 (PMC3610138; doi:10.1186/1472-6750-13-24)
Supplement: Additional file 2: Figure S2 — SDS-PAGE Analysis of C-His6-VanYn from S. venezuelae recombinant strain growth in 3-L batch fermentor as in Figure 3 main text. Crude extracts of cell samples collected at different times of fermentation: 18 (lane 1), 24 (lane 2), 48 (lane 3), 72 (lane 4), 96 (lane 5), 120 (lane 6), 144 (lane 7), 168 (lane 8) hours. In each lane, samples corresponding to 100 μL of cell culture were loaded. Std reference protein: C-His6-VanYn from E. coli (5 μg, 25 kDa). [file 1472-6750-13-24-S2.pdf]

Additional file 2

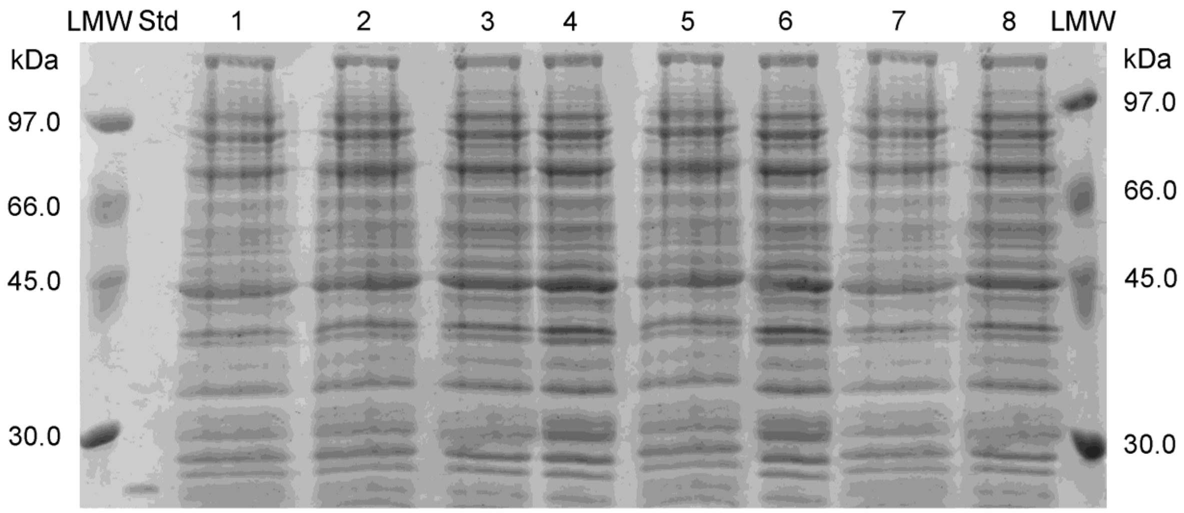

Additional Figure 2

SDS-PAGE Analysis of C-His$_6$-VanY$_n$ from *S. venezuelae* recombinant strain growth in 3-L batch fermentor as in Fig. 3 main text. Crude extracts of cell samples collected at different times of fermentation: 18 (lane 1), 24 (lane 2), 48 (lane 3), 72 (lane 4), 96 (lane 5), 120 (lane 6), 144 (lane 7), 168 (lane 8) hours. In each lane, samples corresponding to 100 µL of cell culture were loaded. Std reference protein: His$_6$-VanY$_n$ from *E. coli* (5 µg, 25 kDa).
